# Tobamoviruses: Special Issue Editorial

**DOI:** 10.3390/v15112174

**Published:** 2023-10-30

**Authors:** Steve Wylie

**Affiliations:** Plant Biotechnology Research Group (Virology), Western Australian State Agricultural Biotechnology Centre, Murdoch University, Murdoch, WA 6150, Australia; s.wylie@murdoch.edu.au

Tobamoviruses are plant-infecting viruses with an ancient lineage, understood to have arisen during the age of the dinosaurs in the Cretaceous period 145–66 million years ago [1]. They are famously resilient, both in terms of their ongoing existence as a group and as tough rod-shaped virions that can remain viable for centuries in dried leaf material [2] and for at least 140,000 years in ice cores from Greenland [3]. Of the forty or so currently described species, a handful are of concern to primary producers of certain crops, notably solanaceous, brassicaceous and cucurbitaceous ones. The first virus to be described was the tobacco mosaic virus (TMV) [4,5,6], the virus that lends its name to the genus. TMV evolved along with its solanaceous hosts in the Americas, and later spread globally on the back of nicotine addiction and a taste for tomatoes and chillis and other solanaceous plants from the New World. Today, other tobamoviruses are spreading globally, and they exact great costs in terms of lost production and the biosecurity efforts required to contain them. Novel tobamoviruses are regularly described, some of which have emerged from wild flora, spreading to cultivated plants.

The first paper in this Special Issue is concerned with the local host range of cucumber green mottle mosaic virus (CGMMV) (https://www.mdpi.com/1999-4915/15/3/683 URL accessed 24 October 2023), a virus of concern to growers of cucurbits (pumpkins, zucchinis, melons, etc). Although CGMMV was first identified in 1935, it has in recent years been identified in an increasing number of counties, including Australia in 2014. Little is known about the transmission of CGMMV between weeds and crop species in Australia. Common weed species belonging to a number of plant families were tested for presence of the virus, and some were inoculated with the virus under laboratory conditions. The samples of six weed species were tested using RT-PCR and RT-qPCR methods, with the latter often detecting CGMMV in samples of *Amaranthus viridis* (family Amaranthaceae). *A. viridis* plants inoculated in the laboratory were infected at a rate of 50–75%, confirming it as an alternative host for CGMMV. Sap taken from infected *A. viridis* plants was inoculated in watermelon seedlings, which became infected and symptomatic. This important study highlighted the importance of controlling *A. viridis* in and around cucurbit crops and called for more research into determining the extent of the CGMMV host range in Australia.

The second paper (https://www.mdpi.com/1999-4915/15/3/728 URL accessed 24 October 2023) focused on the transmission of the tomato brown rugose fruit virus (ToBRFV), a virus that emerged in Israel and surrounding areas in 2015 and is rapidly spreading around the world. ToBRFV remains infectious in plant debris in the soil from previous tomato crops. When a new crop of tomato seedlings is transplanted into infectious soil, they become infected with the virus through wounds in the roots. These authors tested four root-coating formulations aimed at preventing damaged roots from becoming infected with ToBRFV. The four root-coating formulations were methylcellulose, polyvinyl alcohol, silica Pickering emulsion and super-absorbent polymer, all prepared with the disinfectant chlorinated trisodium phosphate. Seedlings were planted in virus-infected soil and tested for ToBRFV infection after a period of time. All control plants with untreated roots became infected. In contrast, all four formulations reduced soil-mediated ToBRFV infection to 0%, 4.3%, 5.5% and 0%, respectively. The tested formulations had no adverse effect on plant growth when compared to untreated control plants grown under virus-free inoculation conditions.

The third paper (https://www.mdpi.com/1999-4915/15/3/743 URL accessed 24 October 2023), also on the topic of CGMMV in Australia, focuses on biosecurity policy. Since the initial incursion of CGMMV into the Northern Territory, detected in 2014, the virus had been detected in three other Australian states despite strict biosecurity restrictions aimed at preventing such spread. The aim of this study was to determine whether new incursions of the virus had occurred from outside sources from contaminated imported seed or whether the initial strain was spreading within the country. All imported cucurbit seed is routinely tested for CGMMV. Plant and pollen samples were collected from across the country and virus genome sequences generated and compared with CGMMV genomes from across the world. All the Australian genomes were tightly grouped together, indicating they most likely originated from one, or possibly two, incursions of very closely related strains, thereby confirming the effectiveness of the biosecurity measures implemented to prevent new CGMMV strains entering Australia.

The fourth paper in the Special Issue (https://www.mdpi.com/1999-4915/15/4/883 URL accessed 24 October 2023) also has an Australian biosecurity focus but examines imported seed batches, which are routinely tested for the presence of a number of tobamoviruses. Of 777 large and small seed lots tested from 2019–2021, 154 carried one or more of five *Tobamovirus* species, including tomato mottle mosaic virus (ToMMV) and ToBRFV, both quarantine pests for Australia. The estimated prevalence of contamination by tobamoviruses ranged from 0.388% to 0.004% in larger contaminated seed lots. In large lots, 20,000 seeds were tested, and this large number aims for a detection confidence level of 99%. The current testing requirement is applied irrespective of the nominal country of consignment origin, recognising that global seed production may involve mixing seed from different jurisdictions. The effectiveness of this approach is that Australia has not recorded an onshore incursion of either ToBRFV or ToMMV.

The final paper (https://www.mdpi.com/1999-4915/15/9/1951 URL accessed 24 October 2023) investigates the use of tobamovirus particles to display antibodies. Transgenic turnip vein clearing virus (TVCV) particles were used as vehicles to display IgG-binding domains D and E of *Staphylococcus aureus* protein A (PA) on every coat protein (CP) subunit to generate TVCV_PA_. Each TVCV particle is made up of hundreds of subunits, and in this case, a single TVCV_PA_ particle displayed up to 500 IgGs, which could be visualised under an electron microscope. The bi-enzyme ensembles of cooperating glucose oxidase and horseradish peroxidase were tethered together on the TVCV_PA_ carriers via a single antibody type, with one enzyme conjugated chemically to its Fc region, and the other one bound as a target, yielding synthetic multi-enzyme complexes. In microtiter plates, the TVCV_PA_-displayed sugar-sensing system were reused in further tests. The engineered virus particles replicated in host plants, and 1–3 mg of TVCV_PA_ particles could be purified per gram of plant tissue. The engineered virus particles are promising candidates for use as adapter coatings on detector chips in biosensor setups applied for electrochemical, label-free real-time analytics. Future work might evaluate the TVCV_PA_ concept not only for bio-detection tasks from food analysis to environmental monitoring but also for in situ signal amplification and biomedical uses. The robust structure of the tobamovirus particle is ideally suited to this application. 

This Special Issue provides a fascinating snapshot of tobamovirus research, from protecting crops with PCR-based, ELISA-based and high-throughput sequencing approaches to phylogenetics and biosecurity polity to exciting research where tobamoviruses are turned into nanoparticles with biomedical applications. I have no doubt that more of these ancient viruses will be discovered in both cultivated and wild plants in the coming decades and centuries, some of which will challenge scientists with the protection of our food supply from them and/or to use their nanometre-sized particles and tiny genomes for exciting new applications.
